# Molecular genetic regulation of the vegetative–generative transition in wheat from an environmental perspective

**DOI:** 10.1093/aob/mcae174

**Published:** 2024-10-04

**Authors:** Tibor Kiss, Ádám D Horváth, András Cseh, Zita Berki, Krisztina Balla, Ildikó Karsai

**Affiliations:** HUN-REN Centre for Agricultural Research, Agricultural Institute, H-2462 Martonvásár, Hungary; Food and Wine Research Institute, Eszterházy Károly Catholic University, H-3300 Eger, Hungary; HUN-REN Centre for Agricultural Research, Agricultural Institute, H-2462 Martonvásár, Hungary; HUN-REN Centre for Agricultural Research, Agricultural Institute, H-2462 Martonvásár, Hungary; HUN-REN Centre for Agricultural Research, Agricultural Institute, H-2462 Martonvásár, Hungary; HUN-REN Centre for Agricultural Research, Agricultural Institute, H-2462 Martonvásár, Hungary; HUN-REN Centre for Agricultural Research, Agricultural Institute, H-2462 Martonvásár, Hungary

**Keywords:** Adaptation, ageing, ambient temperature, circadian clock, earliness, gibberellin response, heading, light perception, photoperiod, vernalization, wheat

## Abstract

The key to the wide geographical distribution of wheat is its high adaptability. One of the most commonly used methods for studying adaptation is investigation of the transition between the vegetative–generative phase and the subsequent intensive stem elongation process. These processes are determined largely by changes in ambient temperature, the diurnal and annual periodicity of daylength, and the composition of the light spectrum. Many genes are involved in the perception of external environmental signals, forming a complex network of interconnections that are then integrated by a few integrator genes. This hierarchical cascade system ensures the precise occurrence of the developmental stages that enable maximum productivity. This review presents the interrelationship of molecular–genetic pathways (*Earliness per se*, circadian/photoperiod length, vernalization – cold requirement, phytohormonal – gibberellic acid, light perception, ambient temperature perception and ageing – miRNA) responsible for environmental adaptation in wheat. Detailed molecular genetic mapping of wheat adaptability will allow breeders to incorporate new alleles that will create varieties best adapted to local environmental conditions.

## INTRODUCTION

Wheat is the third most important cereal crop worldwide, and is an essential source of human food and animal feed (Shewry and Hey, 2015). Its production range (67°N to 45°S, Feldman, 1995) is characterized by wide macro- and microclimatic variation, to which plants can only adapt through wide genetic diversity (Cockram *et al*., 2007; Distelfeld and Dubcovsky, 2010; Dreisigacker *et al*., 2021). One of the most commonly used methods to investigate adaptation is the study of the transition between the vegetative–generative phase and the subsequent intensive stem elongation process. These processes are determined largely by changes in ambient temperature, the diurnal and annual periodicity of the photoperiod, and the composition of the light spectrum (Bullrich *et al*., 2002; Lewis *et al*., 2008; Hemming *et al*., 2012; Karsai *et al*., 2013; Kiss *et al*., 2017; Dixon *et al*., 2019; Monteagudo *et al*., 2020). In temperate cereals, photoperiod and low-temperature vernalization are the two most decisive environmental factors determining the developmental processes of the plant (Cockram *et al*., 2007; Distelfeld *et al*., 2009*a*). In addition, several other factors fine-tune heading or flowering, including the ambient temperature above vernalizing levels and the various characteristics of light. Temperature has a more complex effect than photoperiod on the dynamics of plant development, as it can vary significantly not only seasonally, but also yearly and daily (Bullrich *et al*., 2002; Lewis *et al*., 2008; Hemming *et al*., 2012). Therefore (in addition to its role in regulating the vernalization requirement), temperature significantly affects the heading (used synonymously with flowering) of cereals, the initiation rate, and number of leaves, tillers and spikelets (Slafer and Rawson, 1994; Atkinson and Porter, 1996; Slafer *et al*., 2015). The spectral composition and intensity of light play an important role in the production of both primary and secondary metabolites through photosynthesis. Furthermore, they impact the determination of several developmental parameters, such as flowering time, growing process and the regulation of leaf initiation rate (Chen *et al*., 2004; Darko *et al*., 2014). However, only limited information is available on the relationship between light spectra variation and the complex genetic regulatory mechanism, including the role of circadian rhythm that determines the intensive stem elongation of hexaploid wheat. Results from experiments on barley confirmed that ambient temperature and spectral composition of light strongly modify plant development, even under fully inductive environmental conditions (saturated vernalization requirement and long-day illumination), which are otherwise optimal for differentiation of the floral meristem (Karsai *et al*., 2013). Furthermore, while the genotypic effect of ambient temperature depends on the allelic distribution of the major developmental genes, this correlation was not confirmed for the spectral composition of light (Karsai *et al*., 2013; Monteagudo *et al*., 2020; Del Río *et al*., 2023). A similar response to ambient temperature has been described for wheat (Kiss *et al*., 2017; Dixon *et al*., 2019).

**Figure 1.**
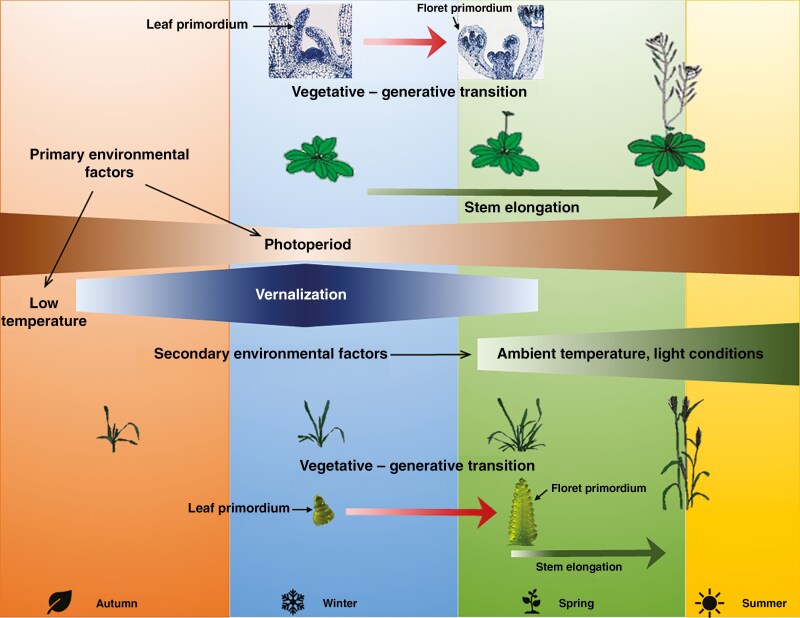
Differences between the generative development of inflorescences and the rate of intensive stem elongation in *Arabidopsis* versus cereals. In *Arabidopsis* these processes occur mostly in parallel, but in cereals they are separated in time; the generative development of the inflorescences is already at advanced stages by the time intensive stem elongation actually starts (Kiss *et al*., 2017; Monteagudo *et al*., 2020).

The molecular genetic regulation of plant development and the transition between the vegetative and generative phases in the dicotyledonous model plant *Arabidopsis thaliana* and in the monocotyledonous genera *Oryza*, *Brachypodium* and *Hordeum* have been studied most extensively (Schaffer *et al*., 1998; Fowler *et al*., 1999; Covington *et al*., 2001; Izawa *et al*., 2002; Yu *et al*., 2002; Karsai *et al*., 2005; Turner *et al*., 2005; Yoo *et al*., 2005; Cockram *et al*., 2007; Higgins *et al*., 2010; Campoli *et al*., 2012*a*, 2012*b*, 2013; Cao *et al*., 2020; Andrade *et al*., 2022). Several known regulatory pathways exist with numerous interconnection points (Levy and Dean, 1998; Mouradov *et al*., 2002; Komeda, 2004; Kim *et al*., 2009; Fornara *et al*., 2010). These pathways include circadian/photoperiod, vernalization (the effect of low temperature), ambient temperature, phytohormones [gibberellic acid (GA)], earliness per se and ageing regulation [micro-RNA (miRNA)]. The signals from the different regulatory elements are collected by a few integrator genes and transmitted to the floral meristem identity genes, which are responsible for the generative transition of the apex and the regular development of the different floral organs, respectively. The molecular genetic process of the developmental phase in wheat is much less well understood, and only the major components of the vernalization and photoperiod regulation pathways have been identified in detail (Worland, 1996; Dubcovsky *et al*., 1998; Worland *et al*., 1998), using either the diploid *Triticum monococcum* and tetraploid species with a smaller genome size or specific crossing lines (RIL – recombinant inbred line, NIL – near-isogenic line, mutant and transgenic lines) (Trevaskis *et al*., 2003; Loukoianov *et al*., 2005; Dubcovsky *et al*., 2006; Yan *et al*., 2006; Shimada *et al*., 2009; Distelfeld and Dubcovsky, 2010; Li *et al*., 2011; Chen and Dubcovsky, 2012; Kumar *et al*., 2012; Kippes *et al*., 2016). Results have revealed significant differences between the regulatory genes and regulatory mechanisms of *Arabidopsis* and cereals, which is particularly striking in the case of vernalization regulation. The genetic regulation of wheat circadian rhythm and GA synthesis is also not well understood, as vernalization and photoperiod responses can mask their effects, making them extremely difficult to study. Also, little information is available on the extent of variability in the phenotypic effects of different alleles as related to heading under field conditions due to the complex interaction of various environmental factors in different years (Kiss *et al*., 2019; Horváth *et al*., 2023). There is also a basic difference, however, between *Arabidopsis* and cereals from aspects of both the generative development of inflorescences and intensive stem elongation (Fig. 1). In *Arabidopsis*, these processes occur in parallel, but in cereals they are separated in time: the generative development of inflorescences is already at advanced stages by the time intensive stem elongation actually starts (Kiss *et al*., 2017; Monteagudo *et al*., 2020).

**Figure 2.**
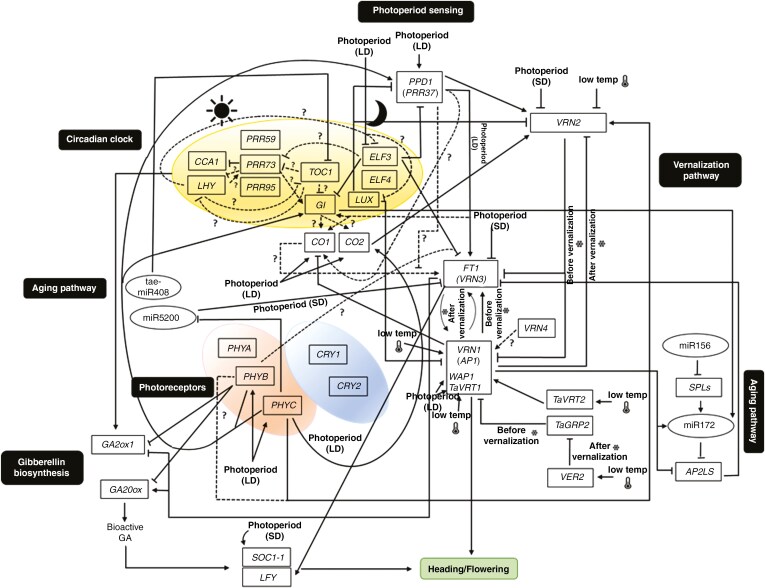
Regulatory relationships of major flowering genes in wheat. Boxes represent genes, while ellipses indicate other genetic factors. Arrows indicate the promotion of gene expression; lines with blunt ends show repression of gene expression. Dotted lines designate probable interactions of the genes presented on the basis of data on interactions of appropriate *Arabidopsis* genes. *Arabidopsis* orthologues of some key flowering genes in wheat are shown in parentheses. LD: long-day illumination, SD: short-day illumination, temp: temperature. The visualized gene regulatory network has been extended based on Cockram *et al*. (2007), Trevaskis *et al*. (2007*b*), Distelfeld *et al*. (2009*a*), Chen and Dubcovsky (2012), Fjellheim *et al*. (2014), Kiseleva and Salina (2018), Cao *et al*. (2021), Debernardi *et al*. (2022) and Li *et al*. (2024). Abbreviations: *AP2L*, *APETALA2-like*; *CCA1*, *CIRCADIAN CLOCK-ASSOCIATED 1*; *CO1*, *CONSTANS 1*; *CO2*, *CONSTANS 2*; *CRY1*, *CRYPTOCHROME 1*; *CRY2*, *CRYPTOCHROME 2*; *ELF3*, *EARLY FLOWERING 3*; *ELF4*, *EARLY FLOWERING 4*; GA, gibberellic acid; GA20ox, GA20 oxidase; GA2ox1, GA2 oxidase 1; *GI*, *GIGANTEA*; *LFY*, *LEAFY*; *LHY*, *LATE-ELONGATED HYPOCOTYL*; *LUX*, *ARRHYTHMO*; miR156, microRNA156; miR172, microRNA172; miR5200, microRNA5200; *PHYA*, *PHYTOCHROME A*; *PHYB*, *PHYTOCHROME B*; *PHYC*, *PHYTOCHROME C*; *PPD1*, *PHOTOPERIOD1*; *PRR59*, *PSEUDORESPONSE REGULATOR 59*; *PRR73*, *PSEUDORESPONSE REGULATOR 73*; *PRR95*, *PSEUDORESPONSE REGULATOR 95*; *SOC1-1*, *SUPPRESSOR OF OVEREXPRESSION OF CONSTANS1-1*; *SPL*, *SQUAMOSA PROMOTER BINDING LIKE*; tae-miR408, *T. aestivum*-microRNA408; *TaGRP2*, *T*. *aestivum glycine-rich RNA binding protein 2*; *TaVRT1*, *Wheat vegetative to reproductive transition-1*; *TaVRT2*, *Wheat vegetative to reproductive transition-1*; *TOC1*, *TIMING OF CAB EXPRESSION1*; *VER2*, *vernalization-related 2*; *VRN1*, *VERNALIZATION1* (*APETALA1*); *VRN2*, *VERNALIZATION2*; *FLOWERING LOCUS T1* (*FT1*), *VRN3* (*VERNALIZATION3*); *VRN4*, *VERNALIZATION4*; *WAP1*, *Wheat APETALA1.*

In summary, genetic regulatory mechanisms that evolved in response to abiotic environmental (vernalization temperature, photoperiod, ambient temperature, light intensity and composition) factors ensure that flowering and ripening occur under optimal environmental conditions. Detailed molecular genetic analysis of wheat heading time may become even more valuable in the future, as rapidly and unpredictably changing macro- and microclimatic influences will increase the need for breeders to find genetic materials in different breeding programmes to produce new varieties that are best adapted to local environmental conditions. This review focuses on the main molecular–genetic regulatory mechanisms responsible for adaptation in wheat (Fig. 2; Table 1).

**Table 1.** Reference list of major genetic regulation networks connected to flowering time in wheat (as a complement to Fig. 2).

Pathways of molecular genetic regulation of plant development	Interaction of genes and other genetic factors	Reference(s)
Vernalization	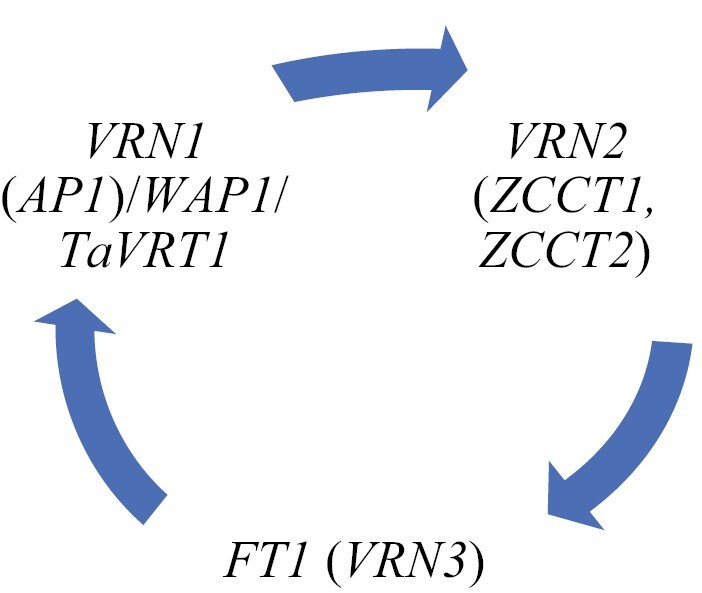	Danyluk *et al*., 2003; Murai *et al*., 2003; Trevaskis *et al*., 2003, 2006, 2007*a*, 2007*b*; Dubcovsky *et al*., 2006; Yan *et al*., 2003, 2004*a*, 2004*b*, 2006; Cockram *et al*., 2007; Kane *et al*., 2007; Shitsukawa *et al*., 2007*a*; Hemming *et al*., 2008; Distelfeld *et al*., 2009*b*; Trevaskis, 2010; Chen and Dubcovsky, 2012; Fjellheim *et al*., 2014; Kiseleva and Salina, 2018; Cao *et al*., 2021; Debernardi *et al*., 2022
	*VRN1* 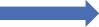 *SOC1-1*, *LFY*	Pearce *et al*., 2013
	*VRN4* 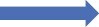 *VRN1* (*AP1*)	Kippes *et al*., 2014; Hyles *et al*., 2020
	*VER2* 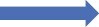 *TaGRP2*	Yong *et al*., 2003; Xing *et al*., 2009; Xiao *et al*., 2014
	*TaGRP2* 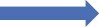 *VRN1* (*AP1*)	Xiao *et al*., 2014
	*TaVRT2* 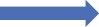 *VRN1* (*AP1*)	Xie *et al*., 2021
Photoperiod	*PPD1* (*PRR37*) 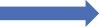 *VRN2* (*ZCCT1*, *ZCCT2*), *FT1* (*VRN3*), *CO1*	Yan *et al*., 2006; Beales *et al*., 2007; Díaz *et al*., 2012; Shaw *et al*., 2012, 2020; Hyles *et al*., 2020; Shaw *et al*., 2020
Circadian clock	*CCA1* 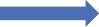 *PRR73*	Kiseleva and Salina, 2018
	*LHY* 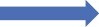 *PRR73*, *ELF3*, *LUX*	Kiseleva and Salina, 2018
	*TOC1* 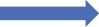 *PRR73*, *LHY*, *GI*	Kiseleva and Salina, 2018
	*GI* 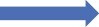 *CO1*/*CO2*, *VRN2* (*ZCCT1*, *ZCCT2*), *FT1* (*VRN3*)	Zhao *et al*., 2005; Li *et al*., 2024
	*CO1*/*CO2* 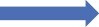 *FT1* (*VRN3*)	Campoli *et al*., 2012*a*; Alqudah *et al*., 2014; Johansson and Staiger, 2014; Mulki and von Korff, 2016; Shaw *et al*., 2020
	*LUX* 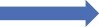 *PPD1* (*PRR37*), *VRN1* (*AP1*), *VRN2* (*ZCCT1*, *ZCCT2*)	Mizuno *et al*., 2016; Nishiura *et al*., 2018
	*ELF3*/*ELF4* 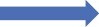 *PPD1* (*PRR37*), *GI*	Yu *et al*., 2008; Alvarez *et al*., 2016, 2023; Zikhali *et al*., 2016
Light perception	*PHYB*, *PHYC* 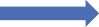 *PPD1* (*PRR37*), *VRN2* (*ZCCT1*, *ZCCT2*)	Chen *et al*., 2014; Pearce *et al*., 2016; Kiseleva and Salina, 2018
Gibberellin response	*GA20ox* 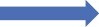 *FT1* (*VRN3*)	Pearce *et al*., 2013
	*GA2ox1* 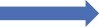 *FT1* (*VRN3*)	
Ageing	tae-miR408 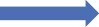 *TOC1*	Zhao *et al*., 2016*b*
	miR5200 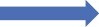 *PHYC*, *FT1* (*VRN3*)	Wu *et al*., 2013; Pearce *et al*., 2016
	miR156 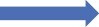 *SPL*s	Debernardi *et al*., 2022
	miR172 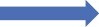 *AP2L*s, *VRN1* (*AP1*), *GI*	Wu *et al*., 2009; Debernardi *et al*., 2022; Li *et al*., 2024
	*AP2L*s 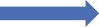 *VRN1* (*AP1*), *FT1* (*VRN3*)	Debernardi *et al*., 2022

## THE MAIN GENETIC DETERMINANTS OF TIME TO HEADING

### Vernalization pathway

Temperate cereals have various mechanisms that can protect the floral meristem from the adverse effects of low temperatures and allow for heading after saturation of the cold requirement (Cockram *et al*., 2007; Brambilla *et al*., 2017; Fernández-Calleja *et al*., 2021). During breeding, mutations in the genes involved in determining vernalization requirement (*VERNALIZATION* – *VRN*) have produced different developmental types of cereal varieties (winter, spring and facultative) with different requirements for low temperatures. Winter types require a longer vernalization period for optimal flowering (Trevaskis, 2010). In wheat, several gene families are involved in the genetic regulation of the vernalization requirement, of which *VRN1* (on the homologous group of chromosome 5), *VRN2* (on 4B, and on the telomeric region of 5A) and *VRN3* (on the homologous group of chromosome 7) have major regulatory roles (Law *et al*., 1976; Snape *et al*., 2001; Barrett *et al*., 2002; Iwaki *et al*., 2002; Galiba *et al*., 2009; Distelfeld and Dubcovsky, 2010). However, the data do not provide a detailed understanding of the environmentally determined interconnections between these gene families in regulating the later developmental phases including the process of stem elongation, beyond their roles in the vegetative–generative transition (Trevaskis *et al*., 2007*a*; Distelfeld *et al*., 2009*b*; Shimada *et al*., 2009; Distelfeld and Dubcovsky, 2010). In barley, different allele combinations of genes that are responsible for the regulation of vernalization requirement and photoperiod sensitivity lead to different plant development types (Karsai *et al*., 2008).

The *VRN1* gene in wheat encodes an important transcription factor of the MINICHROMOSOME MAINTENANCE1/AGAMOUS/DEFICIENS/SERUM RESPONSE FACTOR (MIKC) family of MADS-box genes, which most closely resembles the *APETALA1*/*FRUITFULL* class of *Arabidopsis* MADS-box genes (*AP1*/*FUL*) (Yan *et al*., 2003; Hyles *et al*., 2020). These genes are important for regulating the transition from the vegetative shoot apex to the generative phase (Mandel and Yanofsky, 1995; Ferrándiz *et al*., 2000; Shitsukawa *et al*., 2007*b*). In contrast to *Arabidopsis AP1*/*FUL* genes, the expression of *VRN1* in wheat is increased by prolonged exposure to cold (Danyluk *et al*., 2003; Murai *et al*., 2003; Trevaskis *et al*., 2003; Yan *et al*., 2003). However, the precise mechanism of the low-temperature induction of *VRN1* is not yet understood, but various modifications of histone proteins (H3K27 and H3K4 – DNA methylation) are presumed to play a prominent role. These protein modifications both maintain the repressed state of *VRN1* before winter and also induce increased transcription of this gene after the cold requirement (Diallo *et al*., 2012; Yuan *et al*., 2013; Hyles *et al*., 2020; Chen *et al*., 2023). Oliver *et al*. (2009) found a correlation between histone protein levels and *VRN1* activity. Active histone proteins may be derived from cell organization and may also play a role in cellular ‘vernalization’ memory (Distelfeld *et al*., 2009*a*; Oliver *et al*., 2009; Chen and Dubcovsky, 2012). Transcription of *VRN1* is significantly higher in vernalized plants under short photoperiod than in plants without cold treatment (Dubcovsky *et al*., 2006; Trevaskis *et al*., 2006; Fu *et al*., 2007); therefore, *VRN1* genes are upregulated by vernalization, independently of the function of the other two important *VRN* genes (*VRN2*, *VRN3*) (Trevaskis *et al*., 2007*a*). Similar to *VRN1*, the *WHEAT VEGETATIVE TO REPRODUCTIVE TRANSITION-1* (*TaVRT-1*) and *WHEAT APETALA1* (*WAP1*) genes encode an *APETALA1* (*AP1*)-like MADS-box gene (presumably *VRN1* orthologues). They also play a major role in the vegetative–generative transition of wheat, but they are not sufficient either by themselves or in combination to induce heading (Danyluk *et al*., 2003; Murai *et al*., 2003; Trevaskis *et al*., 2003; Yan *et al*., 2003). Subsequent studies have shown that these two genes are synonymous with *VRN1* (Kane *et al*., 2007; Shitsukawa *et al*., 2007*a*). A number of functional polymorphisms have been found in the promoter, exon and intron regions of *VRN-A1*, including gene copy number variations (Yan *et al*., 2004*a*; Fu *et al*., 2005; Dubcovsky *et al*., 2006; Golovnina *et al*., 2010; Kamran *et al*., 2014; Shcherban *et al*., 2015, 2016; Ivaničová *et al*., 2016; Muterko *et al*., 2016; Muterko and Salina, 2017; Steinfort *et al*., 2017; Strejčková *et al*., 2021; Milec *et al*., 2023; B. Zhang *et al*., 2023). The basic allele type of spring/winter is associated with sequence differences detected in several promoter regions, and a larger insertion/deletion size identified in the intron 1 region (Yan *et al*., 2004*a*; Fu *et al*., 2005). For the other two *VRN1* genes (*VRN-B1* and *VRN-D1*), much less polymorphism was detected, and the spring–winter allele type is basically related to insertion/deletion of the intron 1 region (Yan *et al*., 2004*a*; Fu *et al*., 2005; Santra *et al*., 2009; Golovnina *et al*., 2010; Chu *et al*., 2011; Efremova *et al*., 2011; Milec *et al*., 2012, 2013, 2023; Shcherban *et al*., 2012; J. Zhang *et al*., 2012; Kamran *et al*., 2014; Muterko *et al*., 2015; [Bibr CIT0397][Bibr CIT0339]; Makhoul *et al*., 2022). Copy number variation (CNV) has also been found in the *VRN1* genes, which is particularly significant for *VRN-A1* (Fu *et al*., 2005; Díaz *et al*., 2012; Kippes *et al*., 2015; Würschum *et al*., 2015; Muterko and Salina, 2019, 2021; Strejčková *et al*., 2021). The strength of the correlation between *VRN-A1* copy number and heading time is strongly influenced by the developmental type of each copy. Thus, wheat genotypes that contain more copies of the winter type allele have a much higher vernalization requirement, and they tend to have later heading (Würschum *et al*., 2015; Muterko and Salina, 2019, 2021; Strejčková *et al*., 2021; Chen *et al*., 2024). In relation to the transcriptional expression of *VRN1* genes, Loukoianov *et al*. (2005) found that the three *VRN1* genes (*VRN-A1*, *VRN-B1* and *VRN-D1*) showed different expression levels in isogenic wheat lines between the one- and six-leaf developmental stages. The transcription of *VRN-A1* was already expressed in the first leaf stage, whereas the activity of *VRN-B1* and *VRN-D1* alleles was only detectable in the second and third leaf stages, which may explain its stronger impact on regulation (Loukoianov *et al*., 2005). This phenomenon was confirmed to be the result of the different attributes of dominant/recessive allele types associated with the distinct mutations in each subgenome (promoter insertion in genome A, intron deletions of different lengths in genomes B and D) (Trevaskis *et al*., 2003; Kippes *et al*., 2018; Hyles *et al*., 2020). It was also established that *VRN1* expression follows a diurnal pattern, but little information is available on its variability across genotypes or on its dependence on various environmental factors and whether these variations may have any phenotypic consequences (Shimada *et al*., 2009; Nishiura *et al*., 2014, 2018). The dominant *Vrn-A1* allele also determines the spring type that requires no cold treatment at all for heading. In contrast, the dominant *Vrn-B1*, *Vrn-D1* and *Vrn4* genes only partially abolish the cold requirement that is essential for the generative phase (Pugsley, 1971, 1972; Kato *et al*., 2001; Loukoianov *et al*., 2005).

Two similar ‘zinc-finger CCT’ genes (*ZCCT1* and *ZCCT2*) have been identified in the *VRN2* locus which are involved in dominant flowering-inhibitory mechanisms (Yan *et al*., 2004*b*). No *VRN2* orthologue gene has yet been found either in rice or in *Arabidopsis*, so it appears that this gene is a distinct regulatory element appearing during the evolution of cereals (Yan *et al*., 2004*b*). During vernalization, a steady decrease in the levels of transcription factors produced by *ZCCT1* and *ZCCT2* was detected in the leaves, while the activity of *ZCCT* genes remained high in winter types that were kept at room temperature as controls (Yan *et al*., 2004*b*). Short photoperiod inhibits, while long day stimulates *VRN2* expression (Dubcovsky *et al*., 2006; Trevaskis *et al*., 2006). In barley, both phenomic and gene expression analyses have confirmed that *VRN2* appears to be controlled by both *CONSTANS* (*CO*) and *VRN1*, suggesting that this gene is a joint element in photoperiod and vernalization regulatory pathways (Karsai *et al*., 2005, 2006; Mulki and von Korff, 2016). In hexaploid wheat, the phenotypic effects of loss-of-function alleles of *VRN2* are extremely difficult to study because the redundancy across the subgenomes may hide the effect of a single recessive allele. However, the induction of new allelic variants of this gene (Dubcovsky and Dvorak, 2007; Distelfeld *et al*., 2009*b*; Tan and Yan, 2016; Milec *et al*., 2023) may also broaden the adaptive capacity of wheat (through increased genetic diversity) (Hyles *et al*., 2020). Tan and Yan (2016) reported the duplication of the *VRN-B2* gene in hexaploid wheat, but they found no significant effect on flowering time.

Wheat *FLOWERING LOCUS T* (*TaFT1*), identified as *VRN3*, encodes a RAF kinase inhibitor protein (highly similar to the *FT* gene of *Arabidopsis*) that functions as a signal transduction molecule (an integrator of vernalization and photoperiod regulatory pathways) and, as such, it is an essential element of flowering (Yan *et al*., 2006; Shi *et al*., 2019). At least 12 *FT*-like genetic regions have currently been identified in bread wheat and barley (Lv *et al*., 2014; Bennett and Dixon, 2021; Pieper *et al*., 2021), of which *Vrn-B3* is the most well characterized; the dominant allele was found to have a 5295-bp repetitive sequence insertion in the promoter region, which showed a strong correlation with early flowering. The recessive (*vrn-B3*) allele of this gene with the deletion caused late heading (Yan *et al*., 2006). Moreover, Finnegan *et al*. (2018) described that the late heading observed in relation to deletion was associated with a prolonged spikelet initiation phase, which increased the number of spikelets during long days. Shaw *et al*. (2019) reported a weak interaction between *TaFT2* and heading as well as a stronger association with spikelet number in tetraploid wheat. Gauley and Boden (2021) also observed a strong correlation between *FT2* and *PPD1* gene expression and regulation of spikelet number in hexaploid wheat. Gene expression studies revealed that *FT3* orthologous genes of tetra- and hexaploid wheat were upregulated only under short photoperiod, similar to the expression of *HvFT3* (Halliwell *et al*., 2016). Six additional single nucleotide polymorphisms (SNPs) were found in the promoter and intron 1 regions, but these mutations did not cause phenotypic differences between the two main allele types (Yan *et al*., 2006). Chen *et al*. (2013) and Berezhnaya *et al*. (2021) identified two additional allele types at the *VRN3* locus (*VRN-B3b*, *VRN-B3c* and *VRN-B3d*, *VRN-B3e*). Other polymorphisms linked to *VRN3* have also been reported (Bonnin *et al*., 2008; Zikhali *et al*., 2017; Chen *et al*., 2020*a*; Dreisigacker *et al*., 2021; Nishimura *et al*., 2021), but copy number differences have so far only been detected in barley (Nitcher *et al*., 2013; Milec *et al*., 2023). There is a difference in *VRN3* signalling between *Arabidopsis* and wheat. While *FT* in *Arabidopsis* directly transmits the signal to the floral meristem identity genes via *SUPPRESSOR OF OVEREXPRESSION OF CONSTANS1* (*SOC1*), in wheat, *VRN3* is transmitted via *VRN1* expressed in the apical meristem. Luo *et al*. (2024) demonstrated a repressor role for the wheat *SOC1* gene in the vernalization and photoperiod regulatory pathway. Kiss *et al*. (2017) showed that, with the exception of *VRN3*, ambient temperature also has a significant effect on the expression of the main developmental genes (*VRN1*, *VRN2* and *PPD1*), the extent of which can be significantly influenced by daylength.

Little information is available on *VRN4*, and some authors suggest that the dominant allele of *VRN-D4* has a low distribution among hexaploid wheat genotypes (Goncharov, 1998, 2003). The extra *VRN1* gene copy present at the *VRN4* locus (as a probable result of translocation) may be associated with elevated transcription levels of *VRN1*, which reduces the need for vernalization (Kippes *et al*., 2014). The intron polymorphisms (SNPs) observed in this translocation explain the high expression of *VRN1* in genotypes that carry *VRN4* (Hyles *et al*., 2020). As with other *VRN* genes, *VRN-D4* encodes an AP1 protein that shares a high degree of similarity with the *Arabidopsis* meristem identity protein AP1 (Shi *et al*., 2019). Kippes *et al*. (2015) described the Australian origin of *VRN4*, which can be traced back to the ʻGaboʼ variety and has a significant role in adapting to local environmental conditions through its strong spring growing habit (Hyles *et al*., 2020). In wheat, phenotypic values and gene expression patterns have shown that there is a close epistatic interaction between *VRN1*, *VRN2* and *VRN3* making it difficult to clearly identify the primary target gene in the vernalization process (Distelfeld and Dubcovsky, 2010). However, *VRN1* appears to play a key role (Danyluk *et al*., 2003; Trevaskis *et al*., 2003; Yan *et al*., 2003; Shitsukawa *et al*., 2007*b*).

Other vernalization-induced genes have also been described in wheat, such as *Vernalization-related 2* (*VER2*), *Wheat vegetative to reproductive transition-2* (*TaVRT-2*) and *T. aestivum glycine-rich RNA binding protein 2* (*TaGRP2*) (Chong *et al*., 1998; Yong *et al*., 2003; Kane *et al*., 2005; Xing *et al*., 2009; Xiao *et al*., 2014). Prior to vernalization, the protein produced by *TaGRP2* binds to a specific region of *VRN1* and represses the accumulation of its transcript. However, after vernalization, *VER2* expression is increased, and the phosphorylated *VER2* protein coupled with *TaGRP2* protein resolves its inhibitory effect on *VRN1* (Xiao *et al*., 2014). Previous studies have found that the regulation of *TaVRT2* is independent of vernalization and photoperiod sensing pathways and that its gene product accumulates in the vegetative phase, which can be directly linked to the CArG box part of the promoter region of *VRN1* inhibiting the activity of this gene. This effect is further enhanced by *VRN2*. After vernalization, the expression of both genes is inhibited, triggering an increase in *VRN1* activity (Kane *et al*., 2005, 2007). However, a series of subsequent studies showed that the expression of *TaVRT2* and *HvVRT2* (orthologous gene in barley) genes was induced by vernalization (Trevaskis *et al*., 2007*a*; Dubcovsky *et al*., 2008; Winfield *et al*., 2009; Li *et al*., 2018); thus, they are promoters of the vernalization regulatory pathway together with *TaVRN1* (Xie *et al*., 2021). *TaVRT2* expression increases steadily during vernalization and then decreases significantly, which may prevent the detection of its significant effect on flowering (Xie *et al*., 2021).

Based on recent models of the vernalization regulation pathway (Chen and Dubcovsky, 2012; Debernardi *et al*., 2022; Li *et al*., 2024), in autumn, after germination, when the days are still sufficiently long, the active *VRN2* prevents transcription of *VRN3*, which may play an important role in keeping *VRN1* activity low (Trevaskis *et al*., 2006; Hemming *et al.*, 2008). During vernalization, *VRN1* is progressively activated by cold. The increasing amounts of the VRN1 transcription factor inhibit the function of *VRN2* (its protein is directly linked to the promoter region of *VRN2*) and, in parallel, they stimulate *VRN3* (its protein is directly linked to the promoter region of *VRN3*). VRN3 protein thus activated is transported via the phloem to the meristematic tissue of the shoot apex (Trevaskis *et al*., 2006; Distelfeld *et al*., 2009*a*; Deng *et al*., 2015). The increased amount of *VRN3* further enhances the activity of *VRN1* and induces heading (Kardailsky *et al*., 1999; Kobayashi *et al*., 1999; Abe *et al*., 2005; Loukoianov *et al*., 2005; Preston and Kellogg, 2008; Distelfeld and Dubcovsky, 2010; Chen and Dubcovsky, 2012; Fjellheim *et al*., 2014; Johansson and Staiger, 2014; Deng *et al*., 2015) (Fig. 2; Table 1). The flowering regulation model reported by Debernardi *et al*. (2022) was complemented with an ageing regulation pathway. Based on the results of Shaw *et al*. (2020), the model can be further extended by the finding that, in autumn, *PPD1*, *CO1* and *CO2* genes promote the expression of *VRN2*, and after vernalization, the activated *VRN1* downregulates *VRN2* and *CO1*, promoting the expression of *FT1*, a process that is further enhanced by the *PPD1* gene. In barley, the possible photoperiod-dependent relationship between *PPD-H1* and *VRN-H1* was confirmed by phenomic studies (Karsai *et al*., 1997; Parrado *et al*., 2023).

In summary, while there is a similarity in the genetic regulatory mechanism of the vernalization requirement in mono- and dicotyledonous plants (based on epigenetic histone modification of the promoter of the regulated gene), there is a large difference between the target genes. The main regulatory gene for these processes in *Arabidopsis* is *FLOWERING LOCUS C* (*FLC*), which acts as a repressor in the transduction of the floral meristem, whereas in wheat, it is *VRN1* demonstrating a central activator role (Sheldon *et al*., 2000; Loukoianov *et al*., 2005; Li and Dubcovsky, 2008).

### Photoperiod sensitivity

To detect diurnal and seasonal changes in daylength, plants have had to develop different adaptive systems, in which the gene clusters responsible for photoperiod sensitivity (*PHOTOPERIOD1*/*PPD1*/), genes regulating the internal circadian rhythm, and primary light-sensing molecules such as phytochromes (*PHY*) and cryptochromes (*CRY*) play an important role (Mizuno and Nakamichi, 2005; Hyles *et al*., 2020). Plants can be divided – according to their photoperiod sensitivity – into long-day, short-day and so-called daylength-neutral classes (Garner and Allard, 1920). Long-day plants, such as wheat, barley, rye and *Arabidopsis*, need a long-day illumination to flower properly, otherwise their development will be stunted (Laurie, 1997). For short-day crops, such as maize and rice, a shorter daylight is sufficient for flowering (Laurie, 1997). In contrast to the genetic control of vernalization processes, the photoperiod regulatory pathway shows a higher level of similarity in both monocotyledonous and dicotyledonous plants, in which the activity of *FT* (*TaFT1* in wheat) has a central regulatory role under inductive illumination (Kardailsky *et al*., 1999; Kobayashi *et al*., 1999) and *CO1*/*CO2* can modulate its activity (Kitagawa *et al*., 2012). In *Arabidopsis*, the wild-type dominant *CO* resulted in late flowering under short days and early flowering under long days. In contrast, in cereals, genotypes containing the recessive *co* caused late flowering under both daylengths (Laurie, 1997). For long-day plants, a so-called daylength-uninfluenced developmental phase has been described after germination, followed by a light-inducible phase (Roberts *et al*., 1988). In the period not affected by light, plants do not sense or cannot respond to different daylengths, so their heading time is not affected. The length of this phase may vary between genotypes. When plants enter the so-called light-inducible developmental phase, different daylengths already affect flowering time, but the response of individual genotypes can vary over a wide range (Roberts *et al*., 1988). In wheat, the gene clusters responsible for the regulation of photoperiod sensitivity (*PPD1*) mainly determine flowering time, along with the *VRN* genes. The wild ancestors of wheat are quantitative long-day plants, which are capable of heading under short days, but this process is greatly accelerated by long illumination (Greenup *et al*., 2009). However, mutations in *PPD1* genes (*PPD-A1*, 2A; *PPD-B1*, 2B; and *PPD-D1*, 2D) during breeding resulted in photoperiod-sensitive (*Ppd-1b* – recessive) and photoperiod-insensitive (*Ppd-1a* – semi-dominant) types (Pugsley, 1966; Law *et al*., 1978; McIntosh *et al*., 2003). In wheat and barley, *PPD1* plays a more important role in the regulation of flowering by photoperiod than *CO1* and *CO2* genes (Alqudah *et al*., 2014; Shaw *et al*., 2020). Several studies have shown that under both long- and short-day illumination, photoperiod-insensitive alleles shortened the time needed to heading (under both controlled and field conditions). However, the photoperiod-sensitive allele significantly delays this process in the short day (Worland *et al*., 1998; Foulkes *et al*., 2004; Jones *et al*., 2017). There is evidence that the initial time and length of the stem elongation phase are also highly dependent on the effect of different alleles of the gene responsible for different photoperiod sensing (Miralles *et al*., 2000; Horváth *et al*., 2023). This developmental phase is critical to the formation of the total number of fertile florets, which is closely linked to grain yields (Fischer, 1985; Slafer and Rawson, 1994; Reynolds *et al*., 2009; García *et al*., 2011; Guo *et al*., 2018).

*PPD1* belongs to the pseudoresponse regulator (*PRR*) gene family, and is known as *PRR37* (Beales *et al*., 2007). In addition, *PPD1* is closely related to the *Arabidopsis PRR7* gene which also plays a role in light and temperature sensing of the circadian rhythm, so *PPD1* may have a similar role in cereals (Beales *et al*., 2007; Greenup *et al*., 2009). Although the regulation of *TaPRR37* in wheat is also dependent on circadian genes, this gene is not part of the transcriptional/translational feedback loop that determines circadian rhythm (Kiseleva *et al*., 2022). In terms of functional polymorphisms, the least information is available on *PPD-A1*; only a few polymorphisms have been described so far in photoperiod-insensitive varieties (Beales *et al*., 2007; Wilhelm *et al*., 2009; Nishida *et al*., 2013*b*; Makhoul *et al*., 2024). Breeders might also use the photoperiod-insensitive allele type *PPD-A1a* to fine-tune the photoperiod sensitivity of wheat at higher latitudes (Lin *et al*., 2021). *PPD-B1* functional polymorphisms are better known than those of *PPD-A1*. It was found that the heading time in substitution lines carrying the single-chromosome photoperiod-insensitive allele 2B was shorter than that of the photoperiod-sensitive allele (Scarth and Law, 1984). In Chinese Spring variety, a point mutation in the exon 3 region has been described, and it has also been shown that a multiplication of the *PRR* gene copy number is behind the photoperiod insensitivity (Díaz *et al*., 2012). These mutations show co-segregation with the early heading phenotype (Beales *et al*., 2007; Díaz *et al*., 2012; Würschum *et al*., 2015, 2018). Cane *et al*. (2013), Langer *et al*. (2014) and Kiss *et al*. (2014*b*) in three multi-varietal wheat panels of distinctly diverse geographical origins (Australian vs. mostly European) have independently demonstrated that there is a strong correlation between *PPD-B1* gene copy number variants and flowering time in a wide genetic pool under field conditions. *PPD-D1* in wheat, which shows similarity to *PPD-H1* in barley, has the strongest influence on photoperiod sensitivity (Laurie *et al*., 1995; Börner *et al*., 1998; Beales *et al*., 2007), but there are significant differences between the two genes (Slafer *et al*., 2023). There is a 2089-bp deletion in the promoter region of the *PPD-D1a* allele, which is characteristic of the photoperiod-insensitive allelic variant of *PPD-D1*. As a result of the deletion, the daily cycle of gene expression is significantly modified, the phenotypic consequence of which is early heading under both short- and long-day illumination (Beales *et al*., 2007). In contrast, for *PPD-H1*, the strongest functional polymorphism is based on an SNP in exon 6 (SNP48) or in the CCT domain (SNP22), which does not induce a shift in the diurnal cycling between insensitive and sensitive alleles, and it results in contrasting phenotypic effects of the two alleles depending on the photoperiod (Turner *et al*., 2005; Jones *et al*., 2008; Slafer *et al*., 2023). It is generally accepted that, in wheat, the strongest genetic influence is exerted by the semi-dominant *Ppd-D1a* allele, followed by the dominant *Ppd-B1a* and *Ppd-A1a* alleles (Blake *et al*., 2009; Díaz *et al*., 2012). The activity of the *PPD-D1* gene variant determining the photoperiod-sensitive allele type follows a daily cycle under both long and short days (Beales *et al*., 2007; Zhao *et al*., 2016*a*). In the early morning hours, gene expression level is very low, then reaches its maximum in the morning hours and finally this level starts to decrease. On the other hand, photoperiod-insensitive allele types do not demonstrate this daily fluctuation, and gene activity constantly shows an elevated level, which is closely related to the increase in the activity of *TaFT1* (*VRN3*) and may also affect the decrease in the peak activity of *TaCO1* (Yan *et al*., 2006; Beales *et al*., 2007; Díaz *et al*., 2012; Shaw *et al*., 2012, 2020; Campoli and von Korff, 2014; Hyles *et al*., 2020; Gauley *et al*., 2024). However, there is little information available on the extent to which different allele types of the *PPD-1* gene regulate floral initiation (Beales *et al*., 2007; Shaw *et al*., 2012, 2013; Boden *et al*., 2015; Gauley and Boden, 2021). It is well known that photoperiod-insensitive allele types significantly reduce spikelet number, floret number and fertility, which are key factors in determining yield potential (Boden *et al*., 2015; Prieto *et al*., 2018*a*; Perez-Gianmarco *et al*., 2019). Gauley *et al*. (2024) identified a bZIP and an ALOG transcription factor that suppress flowering and modulate spikelet number and architecture. This new knowledge may help breeders to increase yield potential (Prieto *et al*., 2018*a*; Perez-Gianmarco *et al*., 2019).

There is also little information available on how environmental factors – other than photoperiod – influence the daily activity rhythms of the two allele variants and how they impact flowering.

### Circadian clock

The regulatory mechanism of the plant circadian rhythm has already been significantly explored in *Arabidopsis*, and many homologous genes have been described in wheat (Peng *et al*., 2015), but only a few of them have been studied in detail (Kiseleva and Salina, 2018). Even less information is available on the internal relationship between the genes responsible for the circadian rhythm, developmental genes and light-sensing receptors in cultivated wheat varieties, and how these gene interactions are influenced by environmental elements, such as ambient temperature and light spectral composition, which fundamentally affect the process of intensive stem elongation. A better understanding of these processes may enhance the ability to manipulate the adaptive capacity of cereals and thereby their productivity and geographical distribution. The circadian rhythm is an internal control mechanism (autonomous oscillator) by which plants coordinate their internal biological processes with the daily changes in temperature and light conditions of the external environment (Johansson and Staiger, 2014; Ford *et al*., 2016). It also participates in the regulation of photosynthesis, carbohydrate synthesis, and biotic and abiotic stress responses (Bläsing *et al*., 2005; Fowler *et al*., 2005; Takeuchi *et al*., 2014; Hyles *et al*., 2020; McClung, 2021). In *Arabidopsis*, the core circadian clock consists of three interconnected major transcriptional/translational feedback loops (negative and positive) that shape the daily rhythm of gene expressions (McClung, 2006, 2021; Hsu and Harmer, 2014). Therefore, morning, daytime and evening/night transcriptional loops can be distinguished (Bendix *et al*., 2015). *CIRCADIAN CLOCK-ASSOCIATED 1* (*CCA1*) and *LATE-ELONGATED HYPOCOTYL* (*LHY*) genes encoding an Myb transcription factor have their maximum expression in the early morning hours (Schaffer *et al*., 1998; Cao *et al*., 2021). Then, expression of the *PSEUDORESPONSE REGULATOR* [*PRR* – *PRR3*, *PRR5* (*PRR59* and *PRR95* in monocotyledons), *PRR7* (*PRR37* and PRR73 in monocotyledons), *PRR9* and *TIMING OF CAB EXPRESSION1* (*TOC*1/*PRR1*)] genes also increases until the evening hours (Farre *et al*., 2005; Takata *et al*., 2010; Cao *et al*., 2021). In the morning, the *CCA1* and *LHY* genes inhibit the expression of the *TOC1* and EC [evening complex ‒ *ARRHYTHMO* (*LUX*), *EARLY FLOWERING 3* (*ELF3*), *EARLY FLOWERING 4* (*ELF4*)] genes. In the late afternoon, the expression of *TOC1* increases, which reduces the expression of *CCA1* and *LHY* (negative feedback loop) and also affects its own function, reducing its activity (Schaffer *et al*., 1998; Wang and Tobin, 1998; Matsushika *et al*., 2000; Strayer *et al*., 2000; Alabadi *et al*., 2001; Hazen *et al*., 2005; Huang *et al*., 2012*a*; Bendix *et al*., 2015). *LUX*, and *ELF3* and *ELF4* show maximum expression in the evening and night respectively (Dixon *et al*., 2011; Herrero *et al*., 2012; Hyles *et al*., 2020; Cao *et al*., 2021) and inhibit the function of *PRR5*, *PRR7*, *PRR9*, *TOC1*, *GIGANTEA* (*GI*) and *NIGHT LIGHT-INDUCIBLE AND CLOCK-REGULATED1* (*LNK1*) genes (Kolmos *et al*., 2009; Dixon *et al*., 2011; Helfer *et al*., 2011; Chow *et al*., 2012; Herrero *et al*., 2012); as a result, inhibition of the *CCA1* and *LHY* genes is reduced and a new daily cycle begins (Johansson and Staiger, 2014). The circadian rhythm also plays a key role in the regulation of daylength sensitivity among both monocotyledonous and dicotyledonous plants, as it affects the expression of *CO*, *FT* and *GI* genes (central regulatory elements) (Fowler *et al*., 1999; Kardailsky *et al*., 1999; Kobayashi *et al*., 1999; Samach *et al*., 2000; Distelfeld *et al*., 2009*a*; Lazaro *et al*., 2015; Li *et al*., 2024). Two homologues of the *CO* gene have already been described in wheat (*TaCO1* and *TaCO2*), but their effect on flowering time is still poorly understood (Chen *et al.*, 2014). The opposite regulation (positive/negative) of *CO* on the expression of *FT* (depending on the photoperiod-insensitive and photoperiod-sensitive alleles of the *PPD1*) was observed in both barley and wheat independently of daylength (Campoli *et al*., 2012*a*; Alqudah *et al*., 2014; Johansson and Staiger, 2014; Mulki and von Korff, 2016; Shaw *et al*., 2020). Daily variation in the expression pattern and level of the *TaCO1* was also described (Shaw *et al*., 2013). A decrease in the expression of this gene was observed after the start of illumination; then from 3 to 6 h after this period, the transcription level showed a continuous rise until 15–18 h. The *TaHD1* gene has been described in wheat as an orthologue of *CO* (Hyles *et al*., 2020). Similar to *CO* in *Arabidopsis*, a daily expression pattern (with a peak value during the day) can be observed in *TaHD1* under long-day illumination. The daily rhythm of the wheat *GI* is significantly influenced by daylength (Zhao *et al*., 2005). It is assumed that the *GI* of wheat may have a positive effect on *CO* and indirectly also on the *FT* gene, which can result in early heading (Zhao *et al*., 2005; Li *et al*., 2024). Li *et al*. (2024) established a complex relationship (a combination of inhibitory and inductive processes) between *GI* and *VRN2*, as the daytime expression of *VRN2* in tetraploid wheat *gi-2* mutant lines differed compared to the wild-type. Furthermore, in the mutant lines, the time required for heading increased significantly compared to the wild-type, a phenomenon that became even more pronounced in the case of the dominant allele type of *VRN2*. Although *TaGI* is homologous to *GI* in *Arabidopsis* and has a similar function in wheat (regulation of photoperiod sensitivity), the regulatory mechanism is different in the two species (Li *et al*., 2024). It was found that expression maximum of wheat *TaPRR59* and *TaPRR95* is in the morning hours, which corresponds to the expression peak value of the homologous gene in rice (Kiseleva *et al*., 2022; Rees *et al*., 2022). Furthermore, the expression pattern of *TaPRR59* and *TaPRR95* resembled the transcription pattern of *PRR5* and *PRR9* in *Arabidopsis* (Kiseleva *et al*., 2022; Rees *et al*., 2022). The expression pattern of *TaPRR73* seems to show similarities to *PRR7* in *Arabidopsis*, which may indicate conservation of the functions of homologous genes between species (inhibition of *LHY*/*CCA1*) (Kiseleva *et al*., 2022). During characterization of *TaTOC1* (*TaPRR1*) identified in wheat, it was found that it has a maximum peak expression value in the evening (Zhao *et al*., 2016*b*; Rees *et al*., 2022), which parallels the daily pattern of activity of the homologous gene in *Arabidopsis* (James *et al*., 2008; Rees *et al*., 2022). Although several haplotypes of this gene have been described, which have been proven to be related to agronomic traits (Zhao *et al*., 2016*b*), the signalling mechanism and interaction system of this gene are not yet known (Kiseleva *et al*., 2022). In wheat, a homologous *LUX* (*WPCL1*) gene has been described as a repressor of *PPD1* and *VRN2* (Mizuno *et al*., 2016). Furthermore, Nishiura *et al*. (2018) demonstrated that the upregulation of *VRN1* expression after vernalization occurred in the absence of *LUX* expression. *VRN1* was upregulated in the *LUX* gene deletion mutant (*exe3*), which resulted in extremely early flowering after vernalization, both in long and short days. Thus, the direct regulation of *VRN1* by the circadian pathways independent of the vernalization pathways has also been identified (Nishiura *et al*., 2018). The wheat *ELF3* gene also exerts an inhibitory effect on *PPD1* during the night period (Alvarez *et al*., 2016, 2023), a phenomenon that was also confirmed in *Brachypodium distachyon* (Woods *et al*., 2023). The mutations described in this gene led to the constitutive upregulation of *PPD1*, which was associated with early heading regardless of daylength. Furthermore, *TaELF3* has a negative effect on *TaGI1* that is consistent with the function of its homologous gene in *Arabidopsis* (Yu *et al*., 2008; Zikhali *et al*., 2016). Wittern *et al*. (2023) showed that *ELF3* is expressed in wheat in the morning period and not in the evening as in *Arabidopsis*, and consequently the role of *ELF3* in circadian rhythm regulation is likely to differ between the two species. Furthermore, the co-expression of *ELF3* and *LUX* is not observed at dusk, which also suggests that the mechanism of the circadian oscillator in wheat might differ from that in *Arabidopsis*. Therefore, *ELF3* is an important regulator of various physiological and developmental processes and its different allele types may help to improve plant adaptation, which will be essential for plant breeding (Zahn *et al*., 2023). In barley, one such promising allele might be the exotic *ELF3* allele type observed by Maurer *et al*. (2016), Herzig *et al*. (2018) and Zahn *et al*. (2023), differing by only one nucleotide from the cultivated *ELF3* allele at the *ELF3* locus and accelerates the rate of plant development compared to the cultivated *ELF3* allele.

### Light perception

The spectral composition of the light perceived by plants depends on altitude, latitude, seasons, and climatic and atmospheric factors. During the day (from dawn to dusk), the spectral energy distribution of sunlight changes, so the quality of light also contributes to the precise determination of daylength regulation and circadian rhythm (Morgan and Smith, 1981; Hyles *et al*., 2020). In *Arabidopsis*, several different types of photoreceptors may be distinguished, such as red and far-red light-sensing phytochromes (*PHY* – *PHYA*, *PHYB*, *PHYC*, *PHYD* and *PHYE*), cryptochromes (*CRY1* and *CRY2*), phototropins (*PHOT1* and *PHOT2*), the LOV domain-containing F-box proteins (*ZEITLUPE*/*ZTL*/, *FLAVIN-BINDING KELCH REPEAT F-BOX 1*/*FKF1*/, and *LOV KELCH PROTEIN 1* and *2/LKP1* and *LKP2*/), blue light sensors and *UVB-RESISTANCE 8* (*UVR8*), which is a special UV-B receptor (Guo *et al*., 1998; Yu *et al*., 2010; Liu *et al*., 2011; Ito *et al*., 2012; Chen *et al*., 2014; Pham *et al*., 2018; Sanchez *et al*., 2020; Cao *et al*., 2021). The most important photoreceptors in wheat are phytochromes (*PHYA*, *PHYB* and *PHYC*) and cryptochromes (*CRY1* and *CRY2*). In *Arabidopsis*, *PHYA*, *PHOT1*, *PHOT2*, *CRY1*, *CRY2*, *FKF1* and *UVR8* have a positive effect on flowering (Valverde *et al*., 2004; Sawa *et al*., 2007; Liu *et al*., 2011; Arongaus *et al*., 2018; Kong and Zheng, 2020), while *PHYB*, *PHYC*, *PHYD*, *PHYE*, *ZTL* and *LKP2* negatively influence this process (Devlin *et al*., 1998, 1999; Schultz *et al*., 2001; Monte *et al*., 2003; Valverde *et al*., 2004; Takano *et al*., 2005; Kim *et al*., 2007; Chen *et al*., 2014). In wheat, by contrast, *PHYC* and *PHYB* play a positive regulatory role in the control of flowering under long-day illumination, while the role of *PHYA* is unknown (Chen *et al*., 2014; Pearce *et al*., 2016; Kiseleva and Salina, 2018). There is also no information on possible wheat homologous genes *PHOT*, *UVR8* and *FKF1* (Cao *et al*., 2021). Kiseleva *et al*. (2022) highlighted the difference in the expression pattern of the *ZTL* and *LKP2* homologous genes of wheat and *Arabidopsis*. In barley, the positive correlation of *HvPhyC* with *HvFT1* promoting the reproductive transition of the floral meristem has already been described, an effect that was independent of the circadian cycle and *HvCO1* (Nishida *et al*., 2013*a*). According to other studies, however (Pankin *et al*., 2014), *HvPhyC* had an effect on the circadian oscillation, and it was also related to *PPD-H1*, thus promoting flowering. Similarly, in diploid wheat (*Triticum monococcum*), *PHYB* and *PHYC* upregulate *PPD1* during long days, which results in early flowering through the activation of *FT1* (*VRN3*) and *CO* (Chen *et al*., 2014; Pearce *et al*., 2016). In *Arabidopsis*, the photoreceptor *PHYB* has been described also to function as a temperature signal transmitter (Jung *et al*., 2016; Legris *et al*., 2016). However, this has not yet been investigated in temperate cereals (Cao *et al*., 2021). The regulatory mechanisms of *TaCRY1* and *TaCRY2* have also not been investigated. In *Arabidopsis*, these two genes also play an important role in proper functioning of the internal oscillator (Yu *et al*., 2010; Sanchez *et al*., 2020). It has been described that *CRY2* positively affects one of the genes responsible for regulating the circadian rhythm (*CO*), which, however, also depends on the photoperiod (Guo *et al*., 1998; Suárez-López *et al*., 2001).

### Ambient temperature perception (thermosensory)

This section summarizes the genetic regulatory mechanism of the optimal environmental temperature (between 17 and 23 °C). Temperatures outside of this range can trigger either developmental responses as was discussed in the section on the vernalization pathway or various stress responses in plants (Porter and Gawith, 1999; Acevedo *et al*., 2009). The latter is not the aim of this review. Slafer and Rawson (1995) described that in wheat, raising the temperature from 10 to 19 °C accelerated reproductive development, whereas temperatures above 19 °C delayed terminal spikelet initiation and reduced the number of spikelet primordia. The average optimum temperature required for grain filling was found to be around 20 °C, but a temperature above 35 °C is harmful (Porter and Gawith, 1999). So lower or higher than optimal temperatures inhibit growth and reproductive development, but this effect is strongly modified by daylength (Hemming *et al*., 2012). A higher ambient temperature (25 °C) was observed to have inhibited reproductive development with non-inductive daylength (especially in the early development phases), but accelerated the reproductive phase with inductive daylength (Rawson and Richards, 1993; Kiss *et al*., 2017). This may be a form of adaptive advantage for temperate plants (Jacott and Boden, 2020). Although an increasing number of detailed results are available on the perception of the cold effect (vernalization) by plants and the molecular genetic mechanism of regulation, the related effects of the ambient temperature (as a secondary environmental factor) have been much less well explored. In *Arabidopsis*, several genes have already been identified to be linked to ambient temperature sensing. These include red and far-red light-sensing phytochromes (*PHY*), blue light-sensing cryptochromes (*CRY*), *UVR8*, genes involved in GA biosynthesis, and *SHORT VEGETATIVE PHASE* (*SVP*), *PRR7*, *PRR9*, *GI*, *CO*, *LUX* and *ELF3* genes (Samach *et al*., 2000; Halliday *et al*., 2003; Valverde *et al*., 2004; Salome and McClung, 2005; Samach and Wigge, 2005; Fernández *et al*., 2016; Findlay and Jenkins, 2016). At low temperatures, the transcriptional repressor *ELF3* forms a complex with the *LUX* and *ELF4* genes preventing the activation of *FT* that is necessary for flowering. In response to an increase in temperature, the *ELF3* protein undergoes a molecular structural change that results in the dissociation of the repressor complex and, consequently, in the derepression of *FT*. The PrD (Prion-like domain) structure provides a molecular switch that allows *ELF3* to alter cell organization in response to temperature changes. In barley, it has already been observed that depending on the allele types of *PPD-H1* and *HvELF3*, 28 °C (compared to 20 °C) delayed or accelerated reproductive development (Ejaz and von Korff, 2017; Zhu *et al*., 2023). In spring barley genotypes that carry the mutant *ppd-H1* allele, expression of *FT1* was inhibited at higher ambient temperatures, and late heading and a reduced number of grains per spike were observed compared to the control. In introgression lines carrying the wild-type *PPD-H1* or the mutant *Hvelf3* allele, on the other hand, floret primordia initiation was accelerated (through the increased activity of the *FT1*), and a higher seed number were also observed. Similarly, in spring wheat, higher ambient temperatures either reduce or do not significantly affect *FT1* expression (Kiss *et al*., 2017; Dixon *et al*., 2018). Furthermore, it was observed that ending of the repressive effect of *ELF3* at a higher ambient temperature (25 °C) involved an increase in the expression of the *GI*, *LUX* and *PRR* genes (Ford *et al*., 2016). Thus, at higher temperatures, *ELF3* can play a central role in the regulation processes of photoperiod-dependent flowering (Ford *et al*., 2016). In wheat, Ochagavía *et al*. (2019) described those differences between alleles of *TaELF3* that resulted in different levels of sensitivity to temperature, according to which precociousness in hexaploid wheat was associated with increased sensitivity to temperature in the late reproductive phase. The same study also showed a temperature-dependent inhibition of *TaGI* controlled by *TaELF3*. Genes involved in vernalization also show a strong interaction with temperature (Kiss *et al*., 2017; Dixon *et al*., 2019). Delayed flowering of winter wheat genotypes (following exposure to higher ambient temperatures during and after cold treatment) was described to be genetically closely linked to *VRN1* (highlighting *VRN-A1*) (Dixon *et al*., 2019). Furthermore, higher ambient temperature (25 °C) led to increased expression of *VRN2* associated with reduced levels of *VRN1* and *FT1* transcripts compared to gene activities at moderate (18 °C) and low (11 °C) temperatures (Kiss *et al*., 2017; Dixon *et al*., 2019). The gene expression values influenced by the ambient temperature observed by the authors were significantly dependent on the photoperiod-insensitive and photoperiod-sensitive allele type of *PPD-D1*. Allele types of *VRN1* also influence the expression of flowering-promoting factors in barley. In the treatments at higher ambient temperature, the inhibition of expression of *FT1* was more pronounced in the winter compared to the spring genotypes, related to a stronger repression of *VRN1* (Ejaz and von Korff, 2017).

### Earliness per se

Allelic variants of ‘*Earliness per se*’ (*Eps*) genes can cause differences in flowering time of a few days, independent of environmental effects (Worland, 1996; Snape *et al*., 2001; Bullrich *et al*., 2002; Zikhali *et al*., 2014). A close relationship was also established with the *PPD* and circadian genes, so their interrelated effect determines the final level of photoperiod sensitivity of a given genotype (Worland, 1996; Hyles *et al*., 2020). The most important function of the *Eps* genes is fine-tuning of flowering time (Hoogendoorn, 1985; Valárik *et al*., 2006; Griffiths *et al*., 2009; Horváth *et al*., 2023). Thus, some allelic variants of these genes may be used in the creation of new genotypes that, with their earliness, can avoid the early summer dry period, thus increasing the productivity of the varieties, and therefore also increasing crop security. ‘Earliness per se’ is a quantitative trait determined by a number of genes with smaller effects (Kato and Wada, 1999). In bread wheat, numerous quantitative trait locus (QTL) analyses have already indicated the existence of genes on most chromosomes that may be associated with earliness (Snape *et al*., 2001; Hanocq *et al*., 2007; Chen *et al*., 2010; Basavaraddi *et al*., 2021). These QTL effects were independent of the effects of the main genes of the *VRN* and *PPD* regulatory pathways. However, Horváth *et al*. (2023) identified several *eps* loci in a wheat association panel during field developmental studies, the detectability of which was closely related to the allele type of *PPD-D1*. In most of the cases, the genes behind these earliness loci are not yet known. However, several examples are available where the gene was cloned and characterized and, in some cases, proved to belong to the circadian clock. Zikhali *et al*. (2016) identified that the gene of the *Eps-3A*^*m*^ locus in *Triticum monococcum* is orthologous to *LUX*/*PCL1* of *Arabidopsis* (Shindo *et al*., 2002; Gawroński and Schnurbusch, 2012; Mizuno *et al*., 2012; Gawroński *et al*., 2014). The photoperiod-insensitive mutant version of this gene was associated with early heading and showed increased expression of the *TmFT*, *Ppd-1*, *WCO1* and *TmHd1* genes, and it was also associated with sensitivity to temperature, similar to the *Eps-A*^*m*^*1* (*T*. *monococcum* 1A^m^) gene (Bullrich *et al*., 2002; Lewis *et al*., 2008; Gawroński *et al*., 2014). Bullrich *et al*. (2002) identified a QTL in a mapping population that was closely linked to a major *Eps* gene (*Eps-A*^*m*^*1*). This is one of the best characterized *Eps* loci that is located on chromosome 1A^m^ of *T*. *monococcum* (Bullrich *et al*., 2002; Valárik *et al*., 2006; Lewis *et al*., 2008; Faricelli *et al*., 2016) and is considered to be an orthologue of *TaELF3* (Alvarez *et al*., 2016; Wang *et al*., 2016). The early allele type of this gene (*Eps-Am1-e*) significantly accelerated the time required for flowering, while the late allele type (*Eps-Am1-l*) significantly delayed it. The different temperature optimum of these two allele types was also described by Bullrich *et al*. (2002) and verified by Appendino and Slafer (2003) in *aestivum* wheats. In hexaploid wheat, Zikhali *et al*. (2014) detected a QTL responsible for earliness on chromosome 1DL (*Eps-D1*, homologous to *Eps-A*^*m*^*1*) of hexaploid wheat, the effect of which was confirmed in NIL populations under both field and controlled conditions. They also identified *TaELF3* as the gene responsible for this effect (Zikhali *et al*., 2016). In those lines that carried the deletion of the *Eps-D1* gene, the expression of *TaELF3* was significantly reduced and the expression of *TaGI* changed compared to the wild-type. The temperature-dependent relationship between the early and late allele type of *Eps-D1* and several yield-related components with an emphasis on the number of fertile florets, leaf development dynamics and heading time was analysed in detail (Prieto *et al*., 2020). *Eps* genes probably affect almost all development phases, such as the vegetative/generative transition, early and late spike differentiation, the stem elongation phase, heading, and spike fertility, which also have a significant role in determining grain yield (Lewis *et al*., 2008; Griffiths *et al*., 2009; Ochagavía *et al*., 2018; Prieto *et al*., 2018b). Buckley *et al*. (2024) found that the deletion of *Eps-D1* affects ageing processes and the protein content of the grains.

### Gibberellin response pathways

Plant height is also a key factor determining environmental adaptation. The regulation mechanism of endogenous GA hormone synthesis plays a prominent role in the formation of the final plant height. Gibberellins are pentacyclic diterpene compounds that stimulate growth, so they play an important role in germination, the stem elongation phase, leaf development, the reproductive developmental phase and the regulation of various environmental stress responses (Olszewski *et al*., 2002; MacMillan *et al*., 2005; Yamaguchi, 2008; Llanes *et al*., 2016). Their effect is manifested in the fact that they degrade the growth-inhibiting DELLA proteins. These proteins are encoded by *GAI*, *RGA*, *RGL1*, *RGL2* and *RGL3* in *Arabidopsis*, by *SLR1* in rice, and by *RHT* in wheat (Peng *et al.*, 1997; Ikeda *et al*., 2001). GA is sensed by the protein receptor GID1 (*GIBBERELLIN-INSENSITIVE DWARF1*), which was first identified in gibberellin-insensitive dwarf mutants of rice (Ueguchi-Tanaka *et al*., 2005). Three GID1 orthologues have been described in *Arabidopsis* (AtGID1a, AtGID1b and AtGID1c) with overlapping functions (Nakajima *et al*., 2006). In AtGID1, the triple mutant in *Arabidopsis* GA sensing did not work, and as a result, the plants became extremely dwarfed. This phenotypic effect did not appear in the single mutants, but it did in the double mutants (Griffiths *et al*., 2006; Iuchi *et al*., 2007; Willige *et al*., 2007). Over the last 10 years, the GA signalling mechanism by the GID1 protein and the details of GA biosynthesis itself have been elucidated through biochemical, genetic and structural analyses in rice and *Arabidopsis* (Ueguchi-Tanaka *et al*., 2005; Griffiths *et al*., 2006; Jiang and Fu, 2007; Murase *et al*., 2008; Hirano *et al*., 2010). Seven different types of enzymes may be highlighted in GA biosynthesis, such as ent-copaly1 diphosphate synthase (CPS), ent-kaurene synthase (KS), ent-kaurene oxidase (KO), ent-kaurenoic acid oxidase (KAO), GA20 oxidase, GA3 oxidase and GA2 oxidase (Yamaguchi, 2006). Orthologues of the genes responsible for the synthesis of these enzymes have already been described in wheat (Spielmeyer *et al*., 2004; Appleford *et al*., 2006; Y. Zhang *et al*., 2007; Khlestkina *et al*., 2010; Huang *et al*., 2012*b*; Tang *et al*., 2019) and their expressions were shown to be tissue- and developmental stage-specific (Huang *et al*., 2012b). The role of GA in flowering appears to be species-dependent as it promotes flower initiation in *Arabidopsis*, but inhibits this process in several perennials (Mutasa-Göttgens and Hedden, 2009). Pearce *et al*. (2013) demonstrated that in wheat, *VRN3* also upregulates GA biosynthesis through *GA20ox* and inhibits the expression of *GA2ox1* through indirect and direct ways. The increased level of GA and the transcriptional activity of *VRN1* upregulate the function of the *SUPPRESSOR OF OVEREXPRESSION OF CONSTANS1-1* (*SOC1-1*) and *LEAFY* (*LFY*) genes, a process required for normal spike development (Pearce *et al*., 2013). In hexaploid wheat, GA signalling factors and DELLA proteins are also encoded by *RHT* (Peng *et al*., 1999). The DELLA genes are located at three homologous loci, *Rht-A1*, *Rht-B1* (formerly *RHT1*) and *Rht-D1* (formerly *RHT2*) on chromosomes 4A, 4B and 4D (Börner *et al*., 1996; Flintham *et al*., 1997; Peng *et al*., 1999; Febrer *et al*., 2009). In the *Rht-B1b* and *Rht-D1b* GA-insensitive (mutant) alleles, degraded proteins are produced that are unable to form the appropriate GA–GID–DELLA protein complex, and as a result, the DELLA protein cannot be degraded, but instead accumulates and inhibits the growing processes (Peng *et al*., 1999; Hyles *et al*., 2020). These mutant alleles reduce the length of the internodes that results in reduced plant height (Keyes *et al*., 1989; Hoogendoorn *et al*., 1990) and can also be associated with reduced leaf size (Allan, 1989; Ellis *et al*., 2004). Although the decrease can be observed between all internodes, the largest absolute decrease is found in the last internode length (Hoogendoorn *et al*., 1990). The shorter last internode allows more assimilates to be used for growth and development of the differentiating spikelet (Youssefian *et al*., 1992), and as a result, more fertile florets can be formed that also increase the potential seed number. Recently, Song *et al*. (2023*a*) identified an *Rht-B1* null mutant in wheat segregation lines where a natural deletion of about 500 kb within the *ZnF-B* gene (encoding a RING-type E3 ligase) was observed. This deletion resulted in semi-dwarf plants with more compact plant architecture and significantly increased grain yield (up to 15.2 %) in field experiments compared to *Rht-B1b* and *Rht-D1b* mutants. Further genetic analyses confirmed that deletion of *ZnF-B* in the absence of *Rht-B1b* and *Rht-D1b* alleles caused the semi-dwarf trait by weakening brassinosteroid (BR) sensing. Hence, *Rht-B1* null mutants can be a promising source for the production of new cultivars with high yield and desired height. Dong *et al*. (2023) reported that *GLYCOGEN SYNTHASE KINASE 3* (*GSK3*) phosphorylates *Rht-B1b* modification through the BR pathway, thus causing reduced plant height. Other authors (Li *et al*., 2010; Chen *et al*., 2020*b*) described that the BR sensing pathway in *Arabidopsis* is also related to the regulation of flowering time; however, the molecular–genetic regulatory mechanism of this process remains unclear. In addition, Cui *et al*. (2023) demonstrated that *GSK3* is in physical contact with *VRN1* and regulates its intracellular accumulation through *VRN3*. Other *RHT*s have also been identified as individual allelic variants of *RHT1* and *RHT2* (*RHT3*, *RHT4*, *RHT5*, *RHT6*, *RHT7*, *RHT8*, *RHT9*, *RHT10*, *RHT11*, *RHT12*, *RHT13*, *RHT14*, *RHT15*, *RHT16*, *RHT17*, *RHT18*, *RHT19*; *RHT20*, *RHT21*, *RHT22*, *RHT23*, *RHT24*, *RHT25*, *RHT26* and *RHT27*); although their exact genetic regulation is not yet sufficiently known, their effect on plant height has already been proven (Flintham and Gale, 1983; Konzak, 1987; Loskutova, 1998; Peng *et al*., 1999, 2011; Ellis *et al*., 2004, 2005; Haque *et al*., 2011; Divashuk *et al*., 2012; Li *et al*., 2012; Bazhenov *et al*., 2015; Chen *et al*., 2015; Lu *et al*., 2015; Würschum *et al*., 2017; Yan and Zhang, 2017; Du *et al*., 2018; Ford *et al*., 2018; Mo *et al*., 2018; Mohan *et al*., 2021; Zhao *et al*., 2021; Song *et al*., 2023*b*; Liu *et al*., 2024). There is also little information available about the mechanism of regulation and the relationship between *RHT* genes and other genetic components responsible for flowering regulation in cereals.

Use of *Rht-B1b* and *Rht-D1b* alleles in new breeding lines (‘Green Revolution’ – 1960/1970s) made it possible to replace the tall, lodging-sensitive varieties in the past with short, less lodging-sensitive varieties. The introduction of these genes resulted in higher average grain yields, due to which those cultivars harbouring any combinations of *Rht* mutant allelles spread rapidly in breeding and cereal production (Gao and Chu, 2020). Combining the adequate alleles of the *RHT* genes is extremely important in breeding to avoid adverse phenotypic effects. It has been described that combining the *Rht-B1b* and *Rht-D1b* alleles with the *RHT8* gene resulted in extremely dwarfed plants with reduced spike fertility rates (Worland and Law, 1986). The allelic combinations of *RHT15* and *RHT1* reduced the grain yield per plant (Zhao *et al*., 2023). Cseh *et al*. (2024) also observed that *Rht-B1* and *Rht-D1* mutants are prone to meiotic aberrations (reduced spike fertility rate) even at optimal temperatures and showed a higher level of sensitivity to heat stress than the taller genotypes. In addition, the reduced fertility may be linked to the reduced recombination level of homologous chromosomes and the frequency of defective chromosome separations. Regarding cereal development, genetic regulation of GA biosynthesis lies in the intricate regulation cascade system of plant development, including the vegetative–generative transitions and the stem elongation phase, which is much less well understood.

### Ageing pathway

In *Arabidopsis*, senescence-related genetic factors are also among the most important elements of flowering regulation (Rehman *et al*., 2023) and several *SQUAMOSA PROMOTER BINDING LIKE* (*SPL*) genes regulated by miRNAs have now been described (Xu *et al*., 2016). In *Arabidopsis*, the concentration of *SPL* transcription factors increases continuously with ageing (Fornara *et al*., 2010). These factors promote flowering and ripening, and during this process they also induce the expression of several transcription factors [*LFY*, *FRUITFULL* (*FUL*) and *SOC1*]. miRNAs include short (21–24 nucleotides) non-coding nucleic acid sequences that participate in gene expression regulation (through mRNA cleavage or translation inhibition) (Bartel, 2009; Chellappan *et al*., 2010). miR156 and miR172 are one of the miRNA families showing the greatest degree of conservation in plants (Rehman *et al*., 2023). SPL proteins correlate negatively with miR156, the level of which is significantly higher in young than in older leaves (Fornara *et al*., 2010), while *SPL* genes activate the expression of miR172 in leaves. An increased level of miR172 inhibits the expression of *APETALA2-like* (*AP2L*) genes (flowering-inhibitory transcription factors) promoting flowering competence (Wu *et al*., 2009; Debernardi *et al*., 2022; Li *et al*., 2024). Furthermore, it was described that the synthesis of miRNAs is a temperature-dependent process (e.g. miR156 and miR169 are upregulated at 16 °C) thus preventing precocious flowering at sub-optimal temperatures (Kim *et al*., 2012; Quiroz *et al*., 2021). Other miRNA families have already been identified in wheat by genome-wide association analysis (Yao *et al*., 2007; Sun *et al*., 2014). However, the functions of the identified miRNAs and their role in genetic regulation of plant development are not yet sufficiently detailed. The wheat miR5200 is homologous to that described in *Brachypodium distachyon* and it also inhibits the expression of *FT1* in short-day conditions (Wu *et al*., 2013; Li *et al*., 2014; Pearce *et al*., 2016). These studies also demonstrated that *PHYC* of wheat has a negative effect on this molecule. An miRNA has also been described in wheat (tae-miR408) associated with flowering time (Zhao *et al*., 2016*b*). This molecule exerts a negative effect on the *TaTOC1* circadian rhythm gene, and as a result, the expression level of *TaFT1* that plays an important role in flowering regulation also increases.

### Why is it so important to study the stages of plant development in wheat?

Vernalization requirement and photoperiod sensitivity are the basic components influencing plant developmental phases in cereals grown under continental conditions. After the saturation of these major developmental factors, there are other components (circadian rhythm, light perception, hormonal regulation and *earliness per se*) that sense subsidiary environmental signals, such as light intensity and ambient temperature. This continuous adjustment to environmental conditions ensures the fine-tuning of adaptive plant growth (Fornara *et al*., 2010). As climatic anomalies (taken here to mean weather conditions that are unusual at any given time of year) increase both in magnitude and in frequency, they affect not only fine-tuning mechanisms but also the major plant developmental responses (Porter and Semenov, 2005). It is therefore essential to establish the extent to which disturbances in plant developmental patterns negatively affect yield formation and to obtain detailed physiological and genetic knowledge on the starting date and length of various plant developmental phases. This will enable breeders to modify both the transition from the vegetative to the generative phase of the genotypes by changing the scale of photoperiod-sensitivity and vernalization requirements and the effectiveness of fine-tuning mechanisms (González *et al*., 2005; Borràs *et al*., 2009; Chen *et al*., 2009, 2010; Maurer *et al*., 2016; Zahn *et al*., 2023). The length of the various developmental phases is an important factor determining the extent to which the yield potential of a genotype may be achieved under a given set of ecological conditions (Slafer and Rawson, 1996; Araus *et al*., 2002; González *et al*., 2005; McMaster, 2005; Borràs *et al*., 2009; Chen *et al*., 2009; Foulkes *et al*., 2011). One such adaptation process is a time shift in the rapid stem elongation phase (Fischer, 1985; Slafer and Rawson, 1994; Reynolds *et al*., 2009; García *et al*., 2011). The later timing of stem elongation helps to avoid frost damage in early spring, whereas earlier maturity helps to avoid hot dry weather during summer. Similarly, the relative duration of any two consecutive phases can be also important in determining the various yield components. A longer vegetative phase generates more biomass (due to the longer nutrient storage period), and an extended stem elongation phase is required to achieve a higher number of fertile florets or spikelets, whereas a longer grain-filling period may lead to increased grain weight in the spikes (Kirby, 1988; Slafer and Rawson, 1996; Miralles and Richards, 2000; Whitechurch and Slafer, 2001, 2002; Araus *et al*., 2002; González *et al*., 2002, 2003, 2005; Kiss *et al*., 2011; Dreccer *et al*., 2014; González-Navarro *et al*., 2015, 2016). The time between first node appearance and the start of rapid stem elongation has a significant effect on the number of reproductive tillers, and a close association was observed between the second half of rapid stem elongation (from the boot stage to heading) and the number of spikelets per spike (Miralles and Richards, 2000; Whitechurch and Slafer, 2001, 2002; Kiss *et al*., 2011, 2014*a*). Guo *et al*. (2018) further subdivided the stem elongation phase into seven sub-phases to investigate their effects on yield components. The most important ﬁnding of their study was the potential strategies for controlling the narrow time windows (sub-phases) during the stem elongation phase to increase ﬂoret fertility and grain number. At the time of stem elongation, it is important that the development of node initiation and elongation of the internodes are appropriate for the yield. However, it is still unclear how these events are spatiotemporally coordinated (Huang *et al*., 2024). This approach to cereal stem development may be traced back to information obtained from diploid barley. The main body axis of barley represents a simple and continuous segmentation of phytomers (apex-derived organ form) wherein both vegetative and reproductive organs coexist at opposite ends (Huang *et al*., 2024). In addition to the phytohormones (such as gibberellin), the *FLOWERING LOCUS T* (*FT*)/*TERMINAL FLOWERING 1* (*TFL1*) family genes also play a crucial role in the formation of plant structure (Taoka *et al.*, 2011; Eshed and Lippman, 2019; McKim, 2020). In barley, the *HvFT1* gene integrates signals from vernalization (*VERNALIZATION 1*, *2* – *VRN-H1*, *VRN-H2*), photoperiod (*PHOTOPERIOD 1* – *PPD-H1*) and circadian (*EARLY FLOWERING 3* – *HvELF3*) regulatory pathways for floral induction (Turner *et al*., 2005; Faure *et al*., 2012; Boden *et al*., 2014; Deng *et al*., 2015). Mutations in these flowering time genes may cause significant changes in both vegetative and reproductive phytomeric iterations (Huang *et al*., 2023). However, little information is available on how phytomer initiation and elongation are coordinated during morphogenesis (Huang *et al*., 2024). The effects of vernalization requirement and photoperiod sensitivity on plant developmental stages are much better known than the effects of secondary environmental elements (ambient temperature, light spectra). Temperature affects each phase, and a higher ambient temperature generally accelerates growth and development rates in crop species (Slafer and Rawson, 1994, 1995; Atkinson and Porter, 1996; Slafer *et al*., 2015). However, it is not clear whether the response to temperature is independent of growth rate and development (Kronenberg *et al*., 2021). In relation to the light spectrum, it should be pointed out that the far-red spectrum increases plant internodal length, petiole length, plant height and gibberellin content, among others (Kurepin *et al*., 2010; Hitz *et al*., 2019). The effect of this spectrum has been studied mainly as shade avoidance responses, which are changes in the growth and development pattern of plants caused by shifts in the light spectrum (in the red:far-red light ratio) caused by neighbouring vegetation (Casal *et al*., 1986). In *Arabidopsis*, the regulation of this process is well documented; however, in wheat, it remains limited (Wille *et al*., 2017). In *Arabidopsis*, sunlight activates *PHYB* and *CRY1* to repress shade avoidance responses. The loss-of-function mutants of these photosensory receptors show shade avoidance responses under full sunlight (Mazzella and Casal, 2001). Warm conditions reduce the activity of *PHYB*, which operates as a temperature sensor and further increases the activities of *PHYTOCHROME INTERACTING FACTOR*s (*PIF4* and *PIF7*) by independent temperature sensing mechanisms (Casal and Fankhauser, 2023).

In summary, a more comprehensive and quantitative understanding of the physiological and genetic determinants of time to heading and the partitioning of time among the phenophases of preflowering development would allow the fine-tuning of adaptation and the optimization of development for maximum yield potential under both present and future conditions. Extending the duration of phase intervals with a decisive influence on yield components without modifying the total time to anthesis has been proposed as a promising breeding tool, but this requires detailed information on the mechanism of the genetic and environmental regulation of the start and duration of various phases and their interactions (Chen *et al*., 2009, 2010). However, variations in environmental parameters in different years under field conditions may produce considerable variability in phenotypic responses, often leading to contradictory results (Snape *et al*., 1985; Worland, 1996; Worland *et al*., 1998; Kato *et al*., 2000). The importance of this research field is also underlined by the fact that neither the changes caused by global climatic change on local climate conditions nor their effects on plant developmental strategies can be exactly predicted (Kiss *et al*., 2011).

## CONCLUSIONS

Studying the genes that determine flowering in *Arabidopsis* provides a good foundation for mapping orthologous genes in wheat. This can be of great help for further dissecting and analysing QTLs and genes identified by different molecular genetic and genomic tools as well as for studying their functions and interactions in cereals. This review has provided an overview of the regulatory system of the most important genes that determine the vegetative–generative transition of wheat shoot apex (Fig. 2; Table 1). These genes may be organized around four main regulatory pathways, such as *earliness per se*, light/photoperiod sensitivity, vernalization (cold requirement), hormonal (GA synthesis) and ageing regulation. The results so far have proven the significant differences between the genes determining the vernalization processes of *Arabidopsis* and crop plants, but the regulatory processes show a high degree of similarity in both mono- and dicotyledonous plants. In wheat, the genetic factors of the vernalization, photoperiod and circadian rhythm control pathways (especially the *VRN1*, *VRN2*, *VRN3*, *PPD1*, *CO* and *GI* genes) integrate signals from the environment (vernalizing temperature, daylength), defining the vegetative–generative transition phase, while the importance of GA synthesis and earliness regulation lies in the fine-tuning of further plant development dependent on a set of partially different environmental factors (ambient temperature, daylength, light intensity, spectral composition of light). This complex process has a fundamental effect on the intensive stem elongation phase, which determines various parameters of grain yield. Several studies have already reported on the major regulatory genes responsible for environmental adaptation in wheat and the role of their allelic variants. These results have been established by experiments with diploid (*Triticum monococcum*) and tetraploid (*Triticum turgidum* subsp*. durum*) species with smaller genome sizes or with specific crossing lines (RIL, NIL, mutant and transgenic lines). However, this review also highlights that the current knowledge of the light and temperature control in the process of wheat heading (especially bread wheat) is not yet comprehensive, with only elements of vernalization and photoperiod control being described in detail. Even less is known about complex regulatory pathways such as the circadian rhythm of wheat and its effect on development, the regulatory pathway of GA, or the genetic regulation of earliness and ageing. These areas are extremely difficult to study because vernalization and photoperiod responses, being the most determining, can hide the effects of other regulatory pathways. However, the number of studies linking new genes to the wheat heading process is increasing year by year, further confirming that this information will be an essential task for the future. Elucidating how the epistatic effects of particular allelic variants in different varieties may influence the expression of particular genes under different environmental conditions and how these may be linked will also be important. In summary, detailed omics-based discovery of wheat’s ability to adapt may become increasingly valuable. Due to rapidly and unpredictably changing climatic effects, the need for breeders to find the genetic materials from which they can produce varieties that are most adaptable to local environmental conditions is increasing. In the future, cultivation of these varieties may ensure adequate quality and quantity of yield.
